# Biofilm behavior of *Tannerella forsythia* strains and S-layer glycosylation mutants

**DOI:** 10.1080/20002297.2017.1325190

**Published:** 2017-05-30

**Authors:** Susanne Bloch, Thomas Thurnheer, Yukitaka Murakami, Georgios N. Belibasakis, Christina Schäffer

**Affiliations:** ^a^ Universität für Bodenkultur Wien

## Abstract

The periodontopathogen *Tannerella forsythia* has a characteristic cell surface (S-) layer modified with a unique *O*-glycan. This structure was analyzed for its role in biofilm formation employing an *in vitro* multispecies biofilm model, into which different *T. forsythia* strains and mutants with a modified cell surface composition were incorporated together with nine other oral species. The influence of the glycosylated *T. forsythia* S-layer on the bacterial composition of the biofilms was analyzed quantitatively using quantitative real-time PCR as well as qualitatively by fluorescence *in situ* hybridization and confocal laser scanning microscopy. It was evident that while changes of the *T. forsythia* cell surface did not affect the quantitative composition of the multispecies consortium, with the exception of *Campylobacter rectus* cell numbers, the localization of *T. forsythia* within the biofilm and its aggregation with *Porphyromonas gingivalis* were changed. Thus, the glycosylated *T. forsythia* S-layer might have relevance for positioning of this species within the biofilm and influence its co-localization with *P. gingivalis* and the prevalence of *C. rectus.* This might further pinpoint a pivotal role of *T. forsythia* cell surface structures in the virulence of this species when interacting with host tissues and immune system, from within or beyond the biofilm.

